# Body Mass Index Has a Nonlinear Association With Postoperative 30-Day Mortality in Patients Undergoing Craniotomy for Tumors in Men: An Analysis of Data From the ACS NSQIP Database

**DOI:** 10.3389/fendo.2022.868968

**Published:** 2022-04-20

**Authors:** Yufei Liu, Haofei Hu, Yong Han, Lunzou Li, Zongyang Li, Liwei Zhang, Zhu Luo, Guodong Huang, Zhan Lan

**Affiliations:** ^1^Neurosurgical Department, The First Affiliated Hospital of Shenzhen University, Shenzhen Second People’s Hospital, Shenzhen, China; ^2^Department of Neurosurgery, Beijing Tiantan Hospital, Capital Medical University, Beijing, China; ^3^Shenzhen University Health Science Center, Shenzhen, China; ^4^Department of Nephrology, The First Affiliated Hospital of Shenzhen University, Shenzhen Second People’s Hospital, Shenzhen, China; ^5^Department of Emergency, The First Affiliated Hospital of Shenzhen University, Shenzhen Second People’s Hospital, Shenzhen, China; ^6^Neurosurgical Department, Hechi People’s Hospital, Hechi, China

**Keywords:** body mass index, brain tumor, craniotomy, underweight, mortality risk, risk overestimation

## Abstract

**Background:**

The association between body mass index (BMI) and mortality is controversial. Thus, the purpose of our research was to survey the association between BMI and postoperative 30-day mortality in brain tumor patients undergoing craniotomy.

**Methods:**

This study analyzed data collected in a multicenter, cross-sectional study that consecutively and nonselectively collected data from a total of 18,642 patients undergoing craniotomy for tumors in the ACS NSQIP from 2012 to 2015. We constructed three linear and non-linear binomial logistic models (the inflection point was set at 18.5) to evaluate the association between BMI and postoperative 30-day mortality, respectively. We also conducted subgroup analyses. Additionally, we compared non-linear models with vs. without interaction with sex.

**Results:**

A total of 17,713 patients were included in this analysis. Of these, 47.38% were male. The postoperative 30-day mortality of the included cases was 2.39% (423/17,713), and the mean BMI was 28.41 ± 6.05 kg/m^2^. The linear logistic models suggested that after adjusting for the covariates, BMI was not associated with postoperative 30-day mortality (OR=0.999; 95% CI: 0.981, 1.017). The non-linear binomial logistic models suggested a nonlinear relationship between BMI and postoperative 30-day mortality. When BMI was < 18.5, we observed a stronger negative association between them after adjusting for covariates; the OR and 95% CI were 0.719, 0.576-0.896. When BMI was > 18.5, the relationship between them was not significant. We also found that a one-unit decrease in BMI for male patients with BMI < 18.5 kg/m^2^ was related to a 34.6% increase in the risk of postoperative 30-day mortality (OR=0.654, 95% CI (0.472, 0.907). There was no significant association between them in male patients with BMI > 18.5 kg/m^2^ or female patients.

**Conclusions:**

This study demonstrates a non-linear relationship between BMI and the risk of postoperative death. Preoperative underweight (BMI < 18.5 kg/m^2^) would increase the risk of postoperative death in male patients (> 18 years old) undergoing craniotomy for brain tumors. Appropriate nutritional management prior to craniotomy for brain tumors may reduce the risk of postoperative 30-day mortality in underweight men.

## Background

Craniotomies are the cornerstone of brain tumor treatment. However, craniotomies for intracranial tumors present significant risks of morbidity and mortality (1). The 30-day mortality, an important indicator of perioperative mortality, provides an effective evaluation of the safety of operations and their risk of postoperative complications (2). The 30-day mortality risk of a diagnostic neurosurgical procedure (e.g., resection or tissue biopsy) for a primary pediatric intracranial tumor is between 1.16% and 1.72%, consistent with contemporary data from European populations (3). With the improvement of living standards and the increase in unhealthy diets, the prevalence of obesity has reached pandemic levels in the last 50 years. Obesity represents a major health challenge because it significantly increases the risk of many diseases (such as type 2 diabetes mellitus, fatty liver disease, and hypertension) as well as several cancers (4, 5). Body mass index (BMI) is widely used to define the desirable weight index and is calculated as weight in kilograms divided by height in meters squared. A previous study found that high BMI was linked to 4 million deaths worldwide (6), and a BMI of 35 or greater was associated with significantly higher all-cause mortality (7).

J-Shaped associations between BMI and most specific causes of death and overall mortality were found in a population-based cohort study of 3.6 million adults in the UK; lower BMI was associated with an increased risk of mortality from neurological causes (8). BMI is associated with mortality from many types of cancers. In recent years, increasing studies have investigated the relationship between BMI and prognosis for various cancers. The predictive value of BMI for survival in brain tumor patients is debatable. Underweight BMIs are associated with worse outcomes following craniotomy for brain metastasis (9).

No association was found between overall survival and BMI for patients with normal weights or those with a BMI > 25 with brain metastases due to breast cancer (10). Higher BMIs were associated with longer overall and progression-free survival in adult glioblastoma multiforme patients who underwent surgery and chemoradiotherapy (11). However, another study suggested that BMI was not associated with survival in newly diagnosed and previously untreated patients with GBM (12). To date, neither studies have performed subgroup analyses nor explored the nonlinear relationship between BMI and postoperative 30-day mortality. In addition, the study was limited by the small sample size. The link between BMI and postoperative 30-day mortality has yet to be explored in patients undergoing craniotomies for brain tumors. Thus, the present study was designed to examine the relationship between BMI and postoperative 30-day mortality in cross-sectional study data from a large U.S. brain tumor population. In addition, this study may provide guidance for clinical practice by clarifying the quantitative relationship between BMI and postoperative 30-day mortality.

## Participants and Methods

### Study Design

The present cross-sectional study utilized data from the American College of Surgeons National Surgical Quality Improvement Program (ACS NSQIP) database, from records between 2012 and 2015. Our independent variable was preoperative BMI. The dependent variable was postoperative 30-day mortality.

### Data Source

The data studied obtained from the ACS NSQIP database originally uploaded by Jingwen Zhang et al. (13) (data from “Sepsis and septic shock after craniotomy: Predicting a significant patient safety and quality outcome measure”; DOI: 10.1371/journal.pone.0235273). The original study was an open-access article distributed under a Creative Commons Attribution License, which permits unrestricted use, distribution, and reproduction in any medium, provided the original author and source are credited. Therefore, these data could be used for secondary analysis without infringement on the authors’ rights.

### Participants

**Figure 1:**
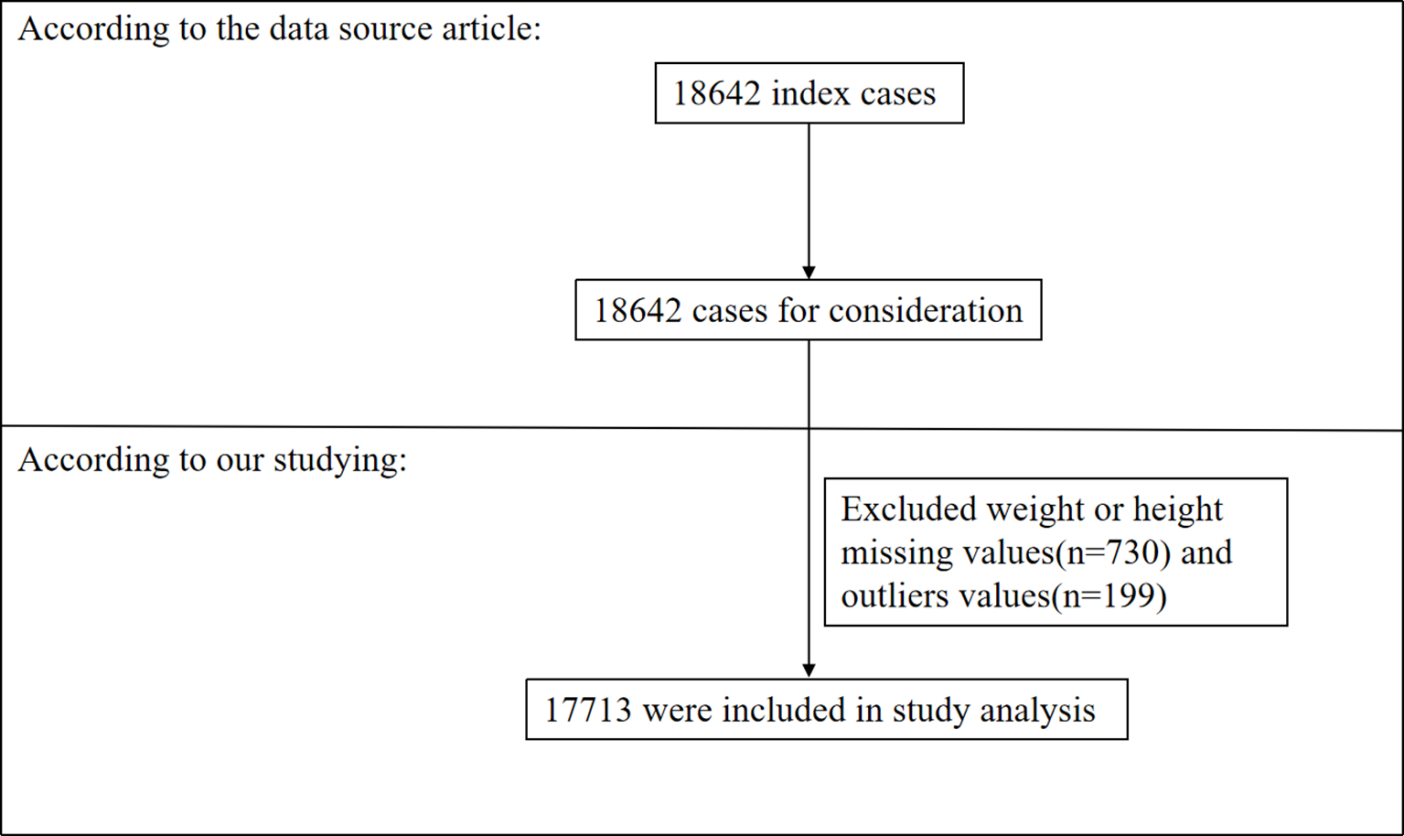
Flowchart of study participant selection.

A total of 18,642 adults with brain tumors were included in the original study. After excluding patients with missing values for weight and/or height (N=730) and outliers (defined as values more than ± 3 standard deviations from the mean) (14) (N=199), 17,713 cases were included in our analysis (as shown in **Figure 1**). Consent forms from participants were not required because our study was based on a secondary analysis of previously collected data and the original personal information was anonymous.

### Variables

#### BMI

Weight and height were recorded as continuous variables. The definition of BMI was as follows: BMI = weight in kilograms divided by height in meters squared (kg/m^2^). The data were collected under standardized conditions and treated following uniform procedures.

### Postoperative 30-Day Mortality

The 30-day mortality was defined as mortality after discharge for the first 30 postoperative days (13).

### Covariates

In our study, covariates were selected according to our clinical experience and the previous literature. Thus, the following variables were treated as covariates: (1) continuous variables: preoperative blood test indicators (serum sodium, blood urea nitrogen, white blood cell count, hematocrit, platelet count), BMI and duration of the operation; and (2) categorical variables: sex (female or male), race (Asian, White, African American or Unknown), age ranges (18-40, 41-60, 60-80, >80 years old), smoking status, ventilator dependence, steroid use, preoperation transfusions, and emergency case status, and a history of diabetes [No, Yes (Noninsulin-dependent) or Yes (Insulin-dependent)], severe chronic obstructive pulmonary disease (COPD), congestive heart failure (CHF), hypertension, renal failure, disseminated cancer, and open wound infection. More specific details are presented in the original study (13). In addition, WBC counts less than or equal to 10 × 10^9^/L were considered low risk, and those greater than 10 × 10^9^/L were considered high risk (15). Thus, the continuous variable WBC count was divided into dichotomous variables (presence of low or high risk) for subgroup analyses.

### Statistical Analysis

We subgroup analyses the participants by quartiles of BMI. The mean ± standard deviation (SD) (normally distributed variables) or median (interquartile range) (non-normally distributed variables) was reported for continuous variables, and frequencies and percentages were presented for categorical variables. We used χ^2^ tests (categorical variables), one-way ANOVAs (normally distributed variables), or Kruskal–Wallis H tests (non-normally distributed data) to test for significant differences among different BMI groups. To examine the exact link between BMI and postoperative 30-day mortality, three distinct covariate unadjusted and multivariable non-linear and linear binomial logistic regression models were constructed according to the STROBE statement guidelines, including a nonadjusted model (no covariates were adjusted), a minimally adjusted model (adjusting for sex, race, age range, smoking status, severe COPD, CHF, and renal failure), and a fully adjusted model (adjusting for sex, race, age range, smoking status, preoperation transfusions, ventilator dependence, severe COPD, congestive heart failure, renal failure, steroid use, serum sodium, blood urea nitrogen, white blood cell count, hematocrit and platelet count). The coefficient of association (OR) and their 95% confidence intervals were recorded. We adjusted the coefficient of association when covariances were added to the model, and the odds ratio was changed by 10% or more (16). BMI < 18.5 kg/m^2^ was defined as underweight according to the World Health Organization. The log-likelihood ratio test was employed to determine the most suitable model for describing the association between BMI and postoperative 30-day mortality. When a nonlinear relationship was found, we set the inflection point of BMI at 18.5, then built a two-piece binary logistic regression model on either side of the inflection point. We also compared non-linear models with vs. without interaction with sex. Tests for interaction were performed with the likelihood ratio test to explore the difference of non-linear relationship between BMI and 30-day mortality in male and female patients.

Subgroup analyses were performed using a binary logistic regression model for the various subgroups: sex, age range, race, diabetes, smoking status, severe COPD, hypertension, disseminated cancer, steroid use, emergency case, and white blood cell count. First, we converted the continuous white blood cell count to a categorical variable based on the clinical threshold mentioned above (<10× 10^9^/L, ≥10 × 10^9^/L) (15). Second, in addition to the subgroup factor itself, we adjusted each subgroup for all factors (race, age range, diabetes, smoking status, ventilator dependence, severe COPD, congestive heart failure, hypertension, renal failure, disseminated cancer, open wound infection, steroid use, preoperative transfusions, emergency case, serum sodium, blood urea nitrogen, white blood cell count, hematocrit, platelet count, and duration of operation). Last, we tested for interaction using the likelihood ratio test for models with and without interaction terms (17, 18).

We also explored the potential for unknown confounds on the relationship between BMI and postoperative 30-day mortality by calculating E-values (19). All results were reported according to the STROBE statement guidelines (16, 20).

The number of participants with missing values for Na, BUN, WBC, HCT, PLT, and duration of operation was 775 (4.37%), 1,415 (7.99%), 575 (3.25%), 428 (2.41%), 565 (3.19%), and 2 (0.01%), respectively. The missing values were replaced by the mean value for statistical analysis.

Modeling was performed with EmpowerStats (http://www.empowerstats.com, X&Y Solutions, Inc., Boston, MA) and the statistical software package R (http://www.R-project.org, The R Foundation). P values < 0.05 (two-sided) were considered statistically significant.

## Results

### Characteristics of Participants

**Table 1** provides the demographic and clinical characteristics of the participants included in the study. A total of 17,713 patients were included in our study, of whom 47.38% were male. The age distribution proportions were 16.4% (18-40), 41.53% (41-60), 38.8% (61-80) and 3.27% (>81). The mean BMI was 28.41 ± 6.05 kg/m^2^. The postoperative 30-day mortality of the included cases was 2.39% (423/17,713). We assigned participants into subgroups using BMI quartiles: Q1 (11.21-24.10 kg/m^2^), Q2 (24.10-27.58 kg/m^2^), Q3 (27.58-31.91 kg/m^2^), and Q4 (31.91-48.90 kg/m^2^). No significant differences were observed among the different BMI groups regarding the covariates of ventilator dependence, renal failure, open wound infection, steroid use, or preoperative transfusions (all P values > 0.05). Compared with those of participants with lower BMI (11.21-24.10 kg/m^2^), the highest BMI (31.91-48.90 kg/m^2^) significantly positively correlated with sex, race, age range, preoperative blood test indicators (serum sodium, blood urea nitrogen, white blood cell count, hematocrit, platelet count), severe COPD, smoking status, congestive heart failure (CHF), hypertension, diabetes, disseminated cancer, emergency case and duration of operation (all P values < 0.05). Although these baseline indicators (such as serum sodium) were statistically significant due to the large sample size, they were not clinically significant.

**Table 1:** Baseline characteristics of participants.

BMI (quartile)	Q1 (11.21-24.10)	Q2 (24.10-27.58)	Q3 (27.58-31.90)	Q4 (31.91-48.90)	P value
N	4410	4441	4429	4433	
Sex N (%)					<0.001
Male	1714 (38.9%)	2359 (53.1%)	2397 (54.1%)	1923 (43.4%)	
Female	2696 (61.1%)	2082 (46.9%)	2032 (45.9%)	2510 (56.6%)	
Race, N (%)					<0.001
White	3094 (70.2%)	3209 (72.3%)	3347 (75.6%)	3354 (75.7%)	
Asian	243 (5.5%)	174 (3.9%)	77 (1.7%)	42 (0.9%)	
African American	256 (5.8%)	246 (5.5%)	293 (6.6%)	408 (9.2%)	
Unknown	817 (18.5%)	812 (18.3%)	712 (16.1%)	629 (14.2%)	
Age range, N (%)					<0.001
18-40	894 (20.3%)	716 (16.1%)	615 (13.9%)	713 (16.1%)	
41-60	1722 (39.0%)	1785 (40.2%)	1807 (40.8%)	2042 (46.1%)	
61-80	1633 (37.0%)	1747 (39.3%)	1849 (41.7%)	1613 (36.4%)	
> 81	161 (3.7%)	193 (4.3%)	158 (3.6%)	65 (1.5%)	
Serum sodium (Mean ± SD)	138.4 ± 3.3	138.6 ± 3.1	138.7 ± 3.1	138.9 ± 3.0	<0.001
Blood urea nitrogen (Mean ± SD)	16.8 ± 7.8	17.5 ± 7.8	17.8 ± 7.7	17.6 ± 8.5	<0.001
White blood cell count (Mean ± SD)	9.2 ± 4.4	9.4 ± 4.4	9.6 ± 4.4	9.7 ± 4.4	<0.001
Hematocrit (Mean ± SD)	39.3 ± 4.8	40.6 ± 4.7	40.9 ± 4.6	40.6 ± 4.6	<0.001
Platelet count (Mean ± SD)	250.7 ± 81.6	239.8 ± 74.0	238.7 ± 70.4	245.7 ± 73.5	<0.001
Duration of operation (Mean ± SD)	202.2 ± 124.7	209.2 ± 134.0	214.6 ± 131.6	227.6 ± 136.1	<0.001
Diabetes, N (%)					<0.001
No	4163 (94.4%)	4058 (91.4%)	3897 (88.0%)	3547 (80.0%)	
Yes (Noninsulin-dependent)	162 (3.7%)	247 (5.6%)	336 (7.6%)	546 (12.3%)	
Yes (Insulin-dependent)	85 (1.9%)	136 (3.1%)	196 (4.4%)	340 (7.7%)	
Smoking status, N (%)					<0.001
No	3261 (73.9%)	3624 (81.6%)	3661 (82.7%)	3734 (84.2%)	
Yes	1149 (26.1%)	817 (18.4%)	768 (17.3%)	699 (15.8%)	
Ventilator dependence, N (%)					0.067
No	4362 (98.9%)	4396 (99.0%)	4372 (98.7%)	4401 (99.3%)	
Yes	48 (1.1%)	45 (1.0%)	57 (1.3%)	32 (0.7%)	
Severe COPD, N (%)					<0.001
No	4120 (93.4%)	4285 (96.5%)	4251 (96.0%)	4264 (96.2%)	
Yes	290 (6.6%)	156 (3.5%)	178 (4.0%)	169 (3.8%)	
Congestive heart failure, N (%)					0.023
No	4396 (99.7%)	4432 (99.8%)	4420 (99.8%)	4410 (99.5%)	
Yes	14 (0.3%)	9 (0.2%)	9 (0.2%)	23 (0.5%)	
Hypertension, N (%)					<0.001
No	3330 (75.5%)	2934 (66.1%)	2535 (57.2%)	2177 (49.1%)	
Yes	1080 (24.5%)	1507 (33.9%)	1894 (42.8%)	2256 (50.9%)	
Renal failure, N (%)					0.057
No	4409 (100.0%)	4441 (100.0%)	4424 (99.9%)	4428 (99.9%)	
Yes	1 (0.0%)	0 (0.0%)	5 (0.1%)	5 (0.1%)	
Disseminated cancer, N (%)					<0.001
No	3146 (71.3%)	3542 (79.8%)	3572 (80.7%)	3673 (82.9%)	
Yes	1264 (28.7%)	899 (20.2%)	857 (19.3%)	760 (17.1%)	
Open wound infection, N (%)					0.221
No	4369 (99.1%)	4408 (99.3%)	4400 (99.3%)	4388 (99.0%)	
Yes	41 (0.9%)	33 (0.7%)	29 (0.7%)	45 (1.0%)	
Steroid use, N (%)					0.058
No	3741 (84.8%)	3797 (85.5%)	3708 (83.7%)	3792 (85.5%)	
Yes	669 (15.2%)	644 (14.5%)	721 (16.3%)	641 (14.5%)	
Preoperation transfusions, N (%)					0.600
No	4391 (99.6%)	4427 (99.7%)	4417 (99.7%)	4419 (99.7%)	
Yes	19 (0.4%)	14 (0.3%)	12 (0.3%)	14 (0.3%)	
Emergency case, N (%)					<0.001
No	4131 (93.7%)	4218 (95.0%)	4201 (94.9%)	4274 (96.4%)	
Yes	279 (6.3%)	223 (5.0%)	228 (5.1%)	159 (3.6%)	
30 day mortality events, N (%)	117 (2.65%)	91 (2.05%)	115 (2.60%)	100 (2.26%)	0.021

### Covariate Unadjusted Analyses Using a Binary Logistic Regression Model

The covariate unadjusted analysis indicated that patients that were female, 41–60 years old, 61–80 years old, >81 years old, had diabetes (Noninsulin-dependent), diabetes (Insulin-dependent), ventilator dependence, severe COPD, congestive heart failure, hypertension, renal failure, disseminated cancer, open wound infections, steroid use, preoperation transfusions, emergency cases, levels of Na, BUN, WBCs, HCT, PLTs and durations of operation were positively associated with postoperative 30-day mortality. In contrast, patients that were Asian, African American, of unknown race and those that smoked were negatively associated with postoperative 30-day mortality (see **Supplementary Table 1** for details).

### Multivariable Analyses Using the Non-Linear and Linear Binomial Logistic Models

To investigate the exact association between BMI and postoperative 30-day mortality, we constructed three models using non-linear and linear binary logistic regression models, respectively. From the linear binary logistic regression models, we found no significant association between BMI and postoperative 30-day mortality rates in Model 1, Model 2, or Model 3 (**Table 2**). The trend of ORs and 95% CIs were robust irrespective of the covariates adjusted for in the model (Model 1: OR=0.987; 95% CI: (0.971, 1.003), Model 2: OR=0.993; 95% CI: (0.975, 1.010), Model 3: OR=0.999; 95% CI: (0.981, 1.017). All p-values for the three standard linear binomial logistic models (Model 1, Model 2, or Model 3) were more than 0.05 (**Table 2**).

**Table 2:** The results of the standard linear and two-piecewise linear regression model.

	Postoperative 30-day mortality (OR, 95% CI, P value)
Model 1 Fitting model by standard linear regression	0.987 (0.971, 1.003) 0.1061
Fitting model by two-piecewise linear regression	
Inflection point of BMI, kg/m^2^	18.5
≤18.5	0.681 (0.553, 0.838) 0.0003
>18.5	0.993 (0.976, 1.009) 0.3917
P for log-likelihood ratio test	0.003
Model 2 Fitting model by standard linear regression	0.993 (0.975, 1.010) 0.3999
Fitting model by two-piecewise linear regression	
Inflection point of BMI, kg/m^2^	18.5
≤18.5	0.716 (0.576, 0.889) 0.0026
>18.5	0.998 (0.981, 1.016) 0.8669
P for log-likelihood ratio test	0.009
Model 3 Fitting model by standard linear regression	0.999 (0.981, 1.017) 0.8998
Fitting model by two-piecewise linear regression	
Inflection point of BMI, kg/m^2^	18.5
≤18.5	0.719 (0.576, 0.896) 0.0033
>18.5	1.005 (0.987, 1.023) 0.5905
P for log-likelihood ratio test	0.010

OR, odds ratios; CI, confidence,

Model 1 (nonadjusted model): not adjusted for any covariates.

Model 2 (minimally adjusted model): adjusted sex, race, age range, smoking status, severe COPD, congestive heart failure, and renal failure.

Model 3 (fully adjusted model): adjusted sex, race, age range, smoking status, ventilator dependence, severe COPD, congestive heart failure, renal failure, steroid use, preoperative transfusions, serum sodium, blood urea nitrogen, white blood cell count, hematocrit, and platelet count.

**Figure 2:**
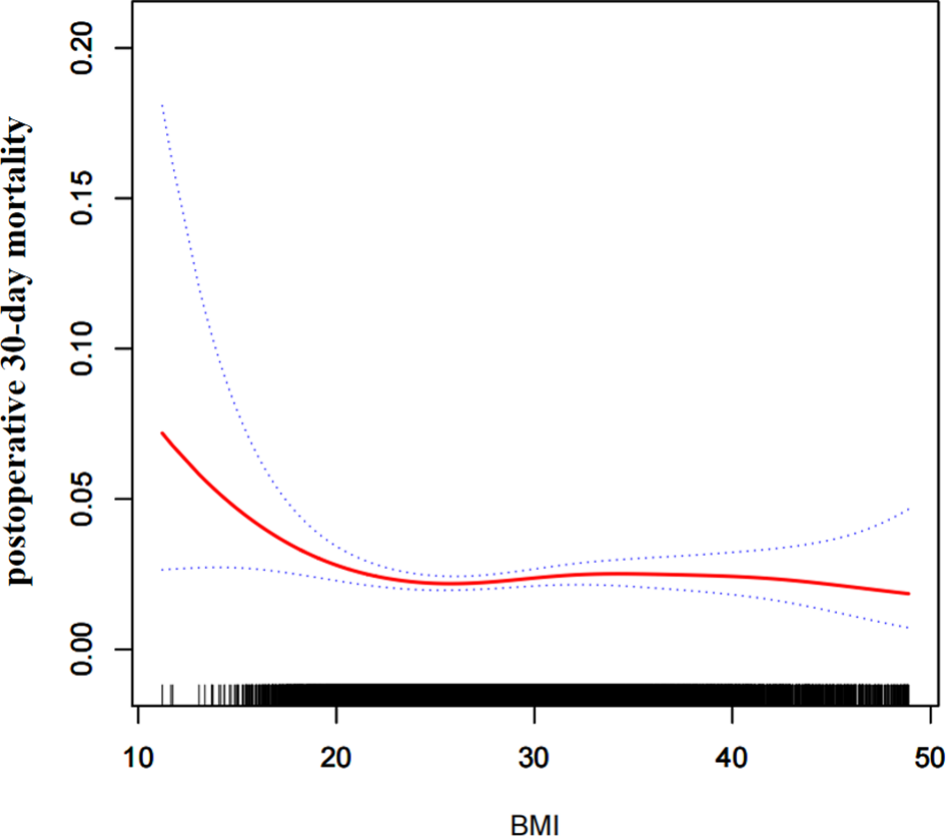
The nonlinear relationship between BMI and risk of postoperative 30-day mortality.

Through the non-linear binary logistic regression models, we observed that the association between BMI and postoperative 30-day mortality rates was nonlinear (**Figure 2**). When the infection point was set at 18.5, the P for the log-likelihood ratio test was less than 0.05 in our study (**Table 2**). After adjusting for confounding factors (sex, race, age range, smoking status, ventilator dependence, severe COPD, congestive heart failure, renal failure, steroid use, preoperative transfusions, serum sodium, blood urea nitrogen, white blood cell count, hematocrit, and platelet count), we observed a stronger negative association between BMI and 30-day mortality. When BMI was less than 18.5, the OR and 95% CI were 0.719, 0.576-0.896, respectively. When BMI was >18.5, the relationship between BMI and 30-day mortality was not significant, the coefficient of association and 95% CI were 1.005, 0.987-1.023, respectively (**Table 2**).

We also computed an E-value to assess the sensitivity to unmeasured confounders. The E-value was 1.08, which was greater than the relative risk of unmeasured confounders influencing the relationship between BMI and postoperative 30-day mortality, suggesting that unmeasured or unknown confounders had little effect on the relationship.

### Subgroup Analyses

We performed subgroup analyses to consider other factors that might influence the relationship between BMI and postoperative 30-day mortality. We used sex, age range, race, diabetes, smoking status, severe COPD, hypertension, disseminated cancer, steroid use, emergency case, and white blood cell count as subgroup variables to detect the trend of coefficient of association. **Table 3** shows that there was no significant differences in the relationship in different age range, race, diabetes, smoking status, severe COPD, hypertension, disseminated cancer, steroid use, emergency case or white blood cell count groups (P for interaction > 0.05); only sex modified the relationship between BMI and postoperative 30-day mortality rates (P for interaction < 0.05). A stronger association was observed in males (OR=0.975, 95% CI: 0.950-1.002), and a weaker association was found in females (OR=1.012, 95% CI: 0.988-1.037).

**Table 3:** Results of interaction and subgroup analyses.

Characteristic	OR (95% CI)	P for interaction
Sex		0.004
Male	0.975 (0.950, 1.002)	
Female	1.012 (0.988, 1.037)	
Age range		0.2003
18-40	1.057 (0.988, 1.131)	
41-60	0.986 (0.956, 1.017)	
61-80	0.999 (0.974, 1.025)	
> 81	0.954 (0.888, 1.024)	
Race		0.6447
White	0.998 (0.977, 1.020)	
Asian	1.023 (0.869, 1.204)	
African American	0.959 (0.896, 1.026)	
Unknown	0.981 (0.937, 1.027)	
Diabetes		0.1382
No	1.001 (0.981, 1.022)	
Yes (Noninsulin-dependent)	0.940 (0.885, 0.998)	
Yes (Insulin-dependent)	0.990 (0.935, 1.048)	
Smoking status		0.4939
No	0.992 (0.972, 1.014)	
Yes	1.008 (0.970, 1.048)	
Severe COPD		0.3361
No	0.997 (0.978, 1.016)	
Yes	0.968 (0.914, 1.026)	
Hypertension		0.1216
No	1.010 (0.982, 1.038)	
Yes	0.981 (0.958, 1.005)	
Disseminated cancer		0.7233
No	0.991 (0.966, 1.016)	
Yes	0.997 (0.971, 1.025)	
Steroid use		0.0944
No	0.984 (0.963, 1.005)	
Yes	1.018 (0.984, 1.052)	
Emergency case		0.4764
No	0.989 (0.970, 1.009)	
Yes	1.011 (0.957, 1.068)	
White blood cell count		0.2992
WBC < 10	1.003 (0.977, 1.030)	
WBC >10	0.984 (0.959, 1.009)	

### Sex Differences in the Nonlinear Relationship

**Figure 3:**
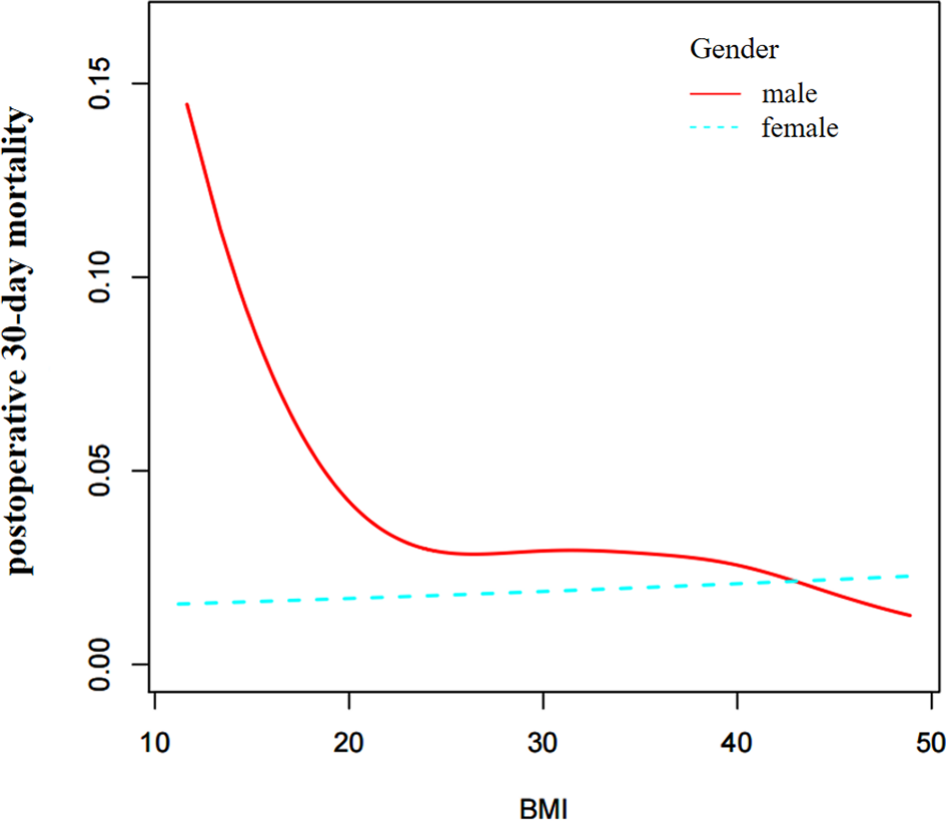
Sex differences in the effect of BMI on postoperative 30-day mortality.

Based on the above results, we further explored the effect of sex on the relationship between BMI and postoperative 30-day mortality. Through the non-linear binary logistic regression models, we found a nonlinear relationship between BMI and postoperative 30-day mortality in males but not in females (**Figure 3**). The mortality rate of male patients was higher than that of female patients (**Figure 3**). As shown in **Table 4**, When the inflection point was set at 18.5, curve fitting and threshold effect analysis revealed that for male patients with BMI < 18.5 kg/m^2^, a one-unit decrease in BMI was related to a 34.6% increase in the risk of postoperative 30-day mortality (OR=0.654, 95% CI (0.472, 0.907); for male patients with BMI > 18.5 kg/m^2^, the effect of a one-unit increase in BMI on risk of postoperative 30-day mortality was not significant. No significant association was found between BMI and postoperative 30-day mortality in female patients (**Figure 3**). We also compared non-linear models with vs. without interaction with sex in **Table 4**. The results of the interaction test were statistically significant, suggesting that the non-linear relationship between BMI and 30-day mortality was different between male and female patients.

**Table 4:** The results of the piecewise linear regression.

Gender	Male		Female	P for interaction
	30-day mortality (OR, 95% CI, P value)		30-day mortality (OR, 95% CI, P value)	
Infection point of BMI	18.5		18.5	
≤18.5	0.654 (0.472, 0.907) 0.0110	≤18.5	0.741 (0.533, 1.029) 0.0738	
>18.5	0.990 (0.964, 1.017) 0.4637	>18.5	1.017 (0.992, 1.042) 0.1803	
P value for log likelihood ratio test	0.022		0.106	<0.001

The model adjusted for race, age range, smoking status, ventilator dependence, severe COPD, congestive heart failure, renal failure, steroid use, preoperative transfusions, serum sodium, blood urea nitrogen, white blood cell count, hematocrit, and platelet count.

## Discussion

Many studies have reported an increased mortality rate in cancer patients with low BMIs (21, 22). Preoperative BMI is an independent prognostic factor for non-small-cell lung cancer patients after surgical resection, with underweight patients having an unfavorable prognosis (23, 24). Additionally, being overweight did not increase the risks of complications and operative mortality compared with those of patients with a normal BMI (24). Another report suggested that underweight BMIs were associated with worse outcomes following craniotomy for brain metastasis (9). These results are similar to ours. We conclude that low BMI is associated with poor outcomes and that being overweight did not increase the risk of postoperative 30-day mortality in patients undergoing craniotomy for tumors.

The predictive value of BMI for survival in many studies is controversial. J-shaped associations between BMI and most specific causes of death and overall mortality were reported in a population-based cohort study of 3.6 million adults in the UK; for neurological causes, a lower BMI was associated with increased mortality risk (8). Based on the results of our study, we share the view that lower BMIs are associated with an increased risk of mortality due to neurological causes. However, our findings are inconsistent with previous findings because higher BMIs also increased mortality in other studies. For instance, a recent study reported that obesity was associated with greater overall mortality in cancer patients, but obese patients with melanoma, renal cell carcinoma, and lung cancer had lower risks of death than patients with the same cancers that were not obese (25). This difference may be attributed to differences in the study population and the cutoff time.

To date, clinical studies on the association between BMI and survival in patients with brain cancer are scarce, and their findings are controversial. Jones LW et al. maintained that there was no association between BMI and survival in newly diagnosed and previously untreated patients with GBM in 2010 (12). Still, Valente Aguiar, P et al. recently reported that a higher BMI was associated with a longer survival index in adult GBM patients who underwent surgery and chemoradiotherapy (11). Lareida A et al. found that underweight BMIs were associated with worse outcomes following craniotomy for brain metastasis (9), which was similar to our findings. These authors speculated that dietary factors might lead to a lower BMI in patients with brain metastasis. In addition, they argued that high BMIs were associated with better outcomes in patients with brain metastases and that improving patient nutrition may help improve their prognosis (9).

To the best of our knowledge, our study was the first to describe a nonlinear relationship between BMI and postoperative 30-day mortality in male patients undergoing craniotomy for tumors. We used a piecewise linear regression model to clarify the nonlinear relationship between BMI and postoperative 30-day mortality. For male patients with BMI < 18.5 kg/m^2^, a one-unit decrease in BMI was related to a 34.6% increase in the risk of postoperative 30-day mortality (OR=0.654, 95% CI (0.472, 0.907). No significant association was found between BMI and postoperative 30-day mortality in male patients with BMI > 18.5 kg/m^2^ or female patients. Other baseline variables might also have influenced patients’ risk of postoperative 30-day mortality. Previously, it was widely believed that obesity, which is usually related to BMI, was associated with reduced survival in cancer patients. However, many studies have challenged this by demonstrating that obesity is associated with improved survival in cancer patients (11, 26). This finding, known as the “obesity paradox”, may be largely explained by methodological limitations, including confounds, selection bias, reverse causation, and reliance on BMI as a measure of adiposity in cancer patients (27, 28). We believe that the BMI may provide an inaccurate measurement of body composition. In general, body habitus is heterogeneous. The distribution of fat, as well as lean body mass and functional performance, may provide more precise insight regarding the association between body habitus and outcomes in cancer patients (29, 30). Moreover, there are differences in the patterns of proportion and distribution of body fat between males and females (31), which may explain the difference in the relationship between BMI and postoperative 30-day mortality between males and females in our findings. Future studies are needed to explore the prognostic significance of quantitative, alternative measures of body fat and functional performance in patients with brain tumors.

Our research has several strengths, as follows: (1) to the best of our knowledge, this is the largest study investigating the relationship between BMI and postoperative 30-day mortality rate, and this large sample size ensures sufficient statistical power; (2) strict statistical adjustments utilized to minimize residual confounds; (3) we used three distinct standard linear and non-linear binomial logistic model**s** GAM model and smooth curve fitting (penalized spline method) to explore the relationship between BMI and postoperative 30-day mortality rate; thus, our analysis had greater clinical value, which previous studies did not explore; (4) we considered the interaction between other covariates and the relationship between BMI and postoperative 30-day mortality because other covariates have the potential to mask the actual relationship between BMI and postoperative 30-day mortality; and (5) the study of coefficient of association facilitates the use of this data in future studies.

However, we also acknowledge that our study has a few limitations, as follows: (1) because this study was a secondary analysis of published data, we cannot exclude some unmeasured and/or residual confounding factors that could influence the estimated relationship (e.g., socioeconomic factors, waist circumference, and waist-to-hip ratio). However, we calculated the E-value to quantify the potential impact of unmeasured confounders and found that they were unlikely to explain the results; and (2) we could not explore the relationship between BMI and long-term outcomes. Our study was based on data from a large and heterogeneous group of patients in a large catchment area despite these limitations. As such, the relationships and conclusions postulated remain highly plausible.

## Conclusions

In patients over 18 years of age who underwent craniotomy for brain tumors, BMI (kg/m^2^) has a specific nonlinear relationship with postoperative 30-day mortality in male patients but not in female patients. Appropriate nutritional management prior to a craniotomy for brain tumors may reduce the risk of postoperative 30-day mortality in underweight men. Thus, our study may provide a reference for policy-makers to develop guidelines as to safer levels of BMI for male patients preparing to undergo craniotomy for brain tumors. Further research on the distribution of adiposity as well as lean body mass in relation to functional performance may provide more accurate insight into the association between body habitus and patient craniotomy outcomes.

## Data Availability Statement

The datasets presented in this study can be found in online repositories. The names of the repository/repositories and accession number(s) can be found in the article/**Supplementary Material**.

## Ethics Statement

The studies involving human participants were reviewed and approved by Ethics Committee of Hechi People’s Hospital. Written informed consent for participation was not required for this study in accordance with the national legislation and the institutional requirements.

## Author Contributions

Formal analysis: YL and HH. Investigation: LL and ZYL. Methodology: YL and HH. Supervision: LZ, ZLu, GH, and ZLa. Writing – original draft: YL, HH, and YH. Writing – review and editing: ZYL, ZLu, and ZLa. All authors contributed to the article and approved the submitted version.

## Conflict of Interest

The authors declare that the research was conducted in the absence of any commercial or financial relationships that could be construed as a potential conflict of interest.

## Publisher’s Note

All claims expressed in this article are solely those of the authors and do not necessarily represent those of their affiliated organizations, or those of the publisher, the editors and the reviewers. Any product that may be evaluated in this article, or claim that may be made by its manufacturer, is not guaranteed or endorsed by the publisher.

## Abbreviations

BMI, body mass index; CI, Confidence Interval; ACS NSQIP, American College of Surgeons National Surgical Quality Improvement Program; SD, standard deviation; COPD, chronic obstructive pulmonary disease; CHF, congestive heart failure; GAM, generalized additive model. GBM, glioblastoma multiforme.
